# Nephritic Factors: An Overview of Classification, Diagnostic Tools and Clinical Associations

**DOI:** 10.3389/fimmu.2019.00886

**Published:** 2019-04-24

**Authors:** Fernando Corvillo, Marcin Okrój, Pilar Nozal, Marta Melgosa, Pilar Sánchez-Corral, Margarita López-Trascasa

**Affiliations:** ^1^Complement Research Group, Hospital La Paz Institute for Health Research (IdiPAZ), La Paz University Hospital, Madrid, Spain; ^2^Center for Biomedical Network Research on Rare Diseases (CIBERER U754), Madrid, Spain; ^3^Department of Medical Biotechnology, Intercollegiate Faculty of Biotechnology, University of Gdansk and Medical University of Gdansk, Gdansk, Poland; ^4^Immunology Unit, La Paz University Hospital, Madrid, Spain; ^5^Pediatric Nephrology Unit, La Paz University Hospital, Madrid, Spain; ^6^Departamento de Medicina, Universidad Autónoma de Madrid, Madrid, Spain

**Keywords:** complement system, nephritic factor, C3 glomerulopathy, lipodystrophy, eculizumab

## Abstract

Nephritic factors comprise a heterogeneous group of autoantibodies against neoepitopes generated in the C3 and C5 convertases of the complement system, causing its dysregulation. Classification of these autoantibodies can be clustered according to their stabilization of different convertases either from the classical or alternative pathway. The first nephritic factor described with the capacity to stabilize C3 convertase of the alternative pathway was C3 nephritic factor (C3NeF). Another nephritic factor has been characterized by the ability to stabilize C5 convertase of the alternative pathway (C5NeF). In addition, there are autoantibodies against assembled C3/C5 convertase of the classical and lectin pathways (C4NeF). These autoantibodies have been mainly associated with kidney diseases, like C3 glomerulopathy and immune complex-associated-membranoproliferative glomerulonephritis. Other clinical situations where these autoantibodies have been observed include infections and autoimmune disorders such as systemic lupus erythematosus and acquired partial lipodystrophy. C3 hypocomplementemia is a common finding in all patients with nephritic factors. The methods to measure nephritic factors are not standardized, technically complex, and lack of an appropriate quality control. This review will be focused in the description of the mechanism of action of the three known nephritic factors (C3NeF, C4NeF, and C5NeF), and their association with human diseases. Moreover, we present an overview regarding the diagnostic tools for its detection, and the main therapeutic approach for the patients with nephritic factors.

## Introduction

**Figure 1:**
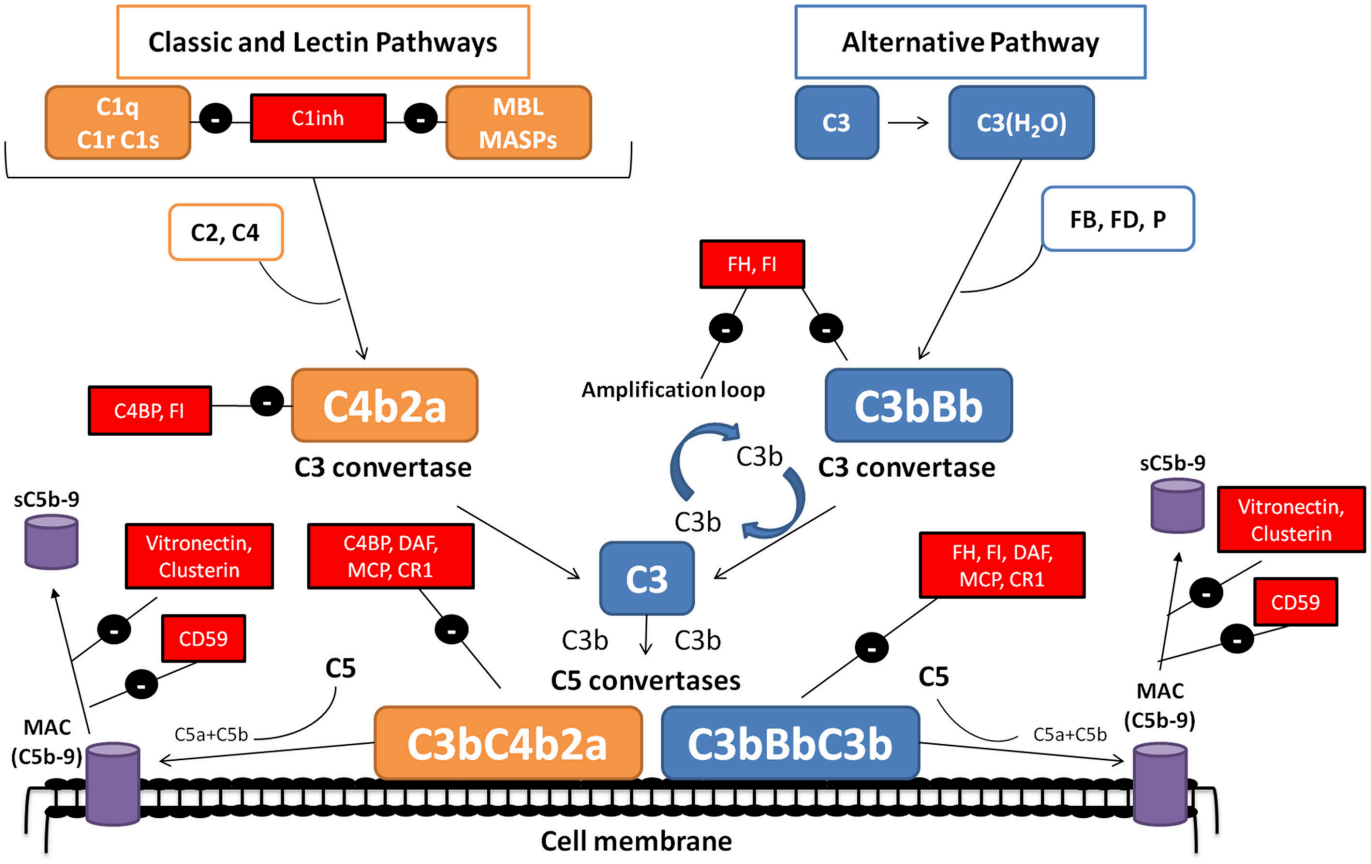
The complement system. The complement system can be activated by three pathways. The classical (CP) and lectin (LP) pathways involve recognition of target-bound antibody or pathogen-specific carbohydrates by C1q or MBL, respectively. In the alternative pathway (AP), continuous, low-level activation of C3 by spontaneous hydrolysis of the internal C3 thioester, or C3 cleavage by plasma proteases, generates C3(H_2_O) or C3b. Activation by any of the three pathways leads to the generation of C3 convertase complexes (C4b2a in the CP/LP and C3bBb in the AP) that cleave C3 into C3a and C3b. Additionally, the AP C3 convertase can bind properdin (P), a positive regulator that stabilizes the enzyme, extending its half-life more than 10-fold. The C3b generated can, in turn, forms more AP C3 convertase, allowing amplification of complement activation. The binding of a new C3b molecule to the C3 convertases creates the C5 convertases (C4b2aC3b or C3bBbC3b), which cleave C5 into C5a and C5b. C5b then initiates the terminal complement pathway, which eventually leads to the formation of the membrane attack complex (C5b-9) and lysis of the target cells. Complement activation is controlled at various levels by different soluble and membrane regulatory proteins (indicated within boxes).

The complement system is a complex molecular system with fundamental roles in apoptotic cell clearance, immune complex elimination, defense against infections, and modulation of adaptive immunity (1). Moreover, complement is capable of distinguishing between self-components and foreign agents. Through a molecular tagging system, complement labels these foreign agents to be eliminated by opsonophagocytosis or by direct cell lysis (2, 3). The complement cascade is activated by three distinct mechanisms: the classical pathway (CP), the lectin pathway (LP) and the alternative pathway (AP) (Figure 1). These three pathways differ mainly in the initial activation steps, but all of them converge in the activation of the C3 molecule through the generation of unstable protease complex, called C3 convertases (1–4).

The activation of CP and LP pathways is triggered by the binding of antigen–antibody complexes, and through the recognition of mannose groups on bacterial surfaces, respectively. After its activation, the resulting multi-molecular complex C3 convertase (C4b2a), is responsible for cleaved C3 molecule into C3a and C3b (Figure 1) (1). The next step concludes with the incorporation of C3b molecules to the C4b2a complex, and the generation of the CP/LP C5 convertase (C4b2aC3b). Finally, C5 convertase splits C5 into C5a, a pro-inflammatory anaphylatoxin, and C5b, which incorporates to the formation of the of the membrane attack complex (MAC or C5b9), along with C6, C7, C8, and C9, on the cell surface. Two soluble proteins, called vitronectin and clusterin, regulate this process, preventing its incorporation into cell membranes (1–4) (Figure 1).

In contrast to the CP and LP, the AP is initiated by spontaneous activation of C3 in plasma, which occurs through the “tick-over” (1, 2). This spontaneous activation results in the production of a few C3a and C3b molecules. The resulting C3b molecules can then combine with factor B (FB) to form the inactive AP pro-C3 convertase (C3bB). Then FB is cleavage by factor D (FD), generating Ba, which is released from the complex, and Bb, which remains bound. The assembled C3bBb complex is considered the functional C3 convertase of the AP, and is capable of amplifying complement activation by a feedback mechanisms that generates a large number of C3b molecules within a short time span (1–4) (Figure 1). There is a complement positive regulator, called properdin (P), which can bind to the C3bBb complex inducing an increase of his half-life (1, 2). Such as happened in CP and LP, generation of the AP C5 convertase (C3bBbC3b) cleaves C5 and ends in MAC formation (1, 2).

The convertase complexes dissociate spontaneously in a few minutes, a process that is critical to prevent autologous tissue injury. To prevent this damage, there is a group of soluble complement regulatory proteins (Factor H (FH), Factor I (FI) and C4BP) and membrane proteins (MCP/CD46, DAF/CD55, CR1/CD35, and CD59) with crucial roles in accelerate C3/C5 convertase dissociation, and/or inactivate C3b by proteolytic cleavage (4, 5) (Figure 1). A strict control between activation and regulation is necessary; as otherwise, the situation can result in the appearance of several diseases. Protein deficiencies or protein abnormal function due to genetic variations, or the presence of autoantibodies against complement components, such as nephritic factors (NFs), are associated with several diseases, including C3 glomerulopathy (C3G), membranoproliferative glomerulonephritis (MPGN), acquired partial lipodystrophy (APL) or Systemic Lupus Erythematosus (SLE) (6–8).

## Nephritic Factors

### C3 and C5 Nephritic Factors

**Figure 2:**
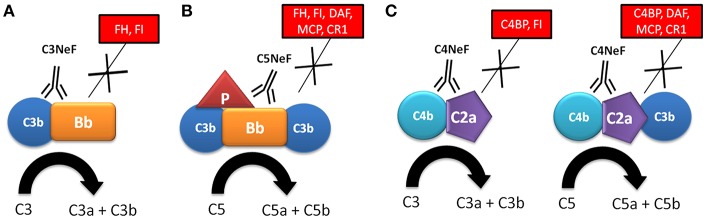
Mechanism of action of nephritic factors. **(A)** C3NeF binds to a neoepitope on the C3 convertase of the alternative pathway (C3bBb), preventing its spontaneous and FH-mediated decay, and increasing its half-life and C3 consumption. **(B)** C5NeF binds to a neoepitope on the C5 convertase of the alternative pathway in the presence of properdin (P) (C3bBbC3bP), preventing its spontaneous and regulator-mediated decay, and increasing its half-life and C5 consumption. **(C)** C4NeF binds to a neoepitope on the C3/C5 convertases of the classical/lectin pathway (C4b2a/C4b2aC3b), preventing its spontaneous and regulator-mediated decay, and increasing its half-life and C3 consumption. Soluble and membrane complement regulators are indicated within boxes.

NFs were first described in 1969, based on the observation that the serum from a patient with MPGN and hypocomplementemia broke down C3 when it was mixed with normal human serum (9, 10). The activity of NFs was later attributed to the stabilization of either cell-bound or fluid phase AP complement convertase by its incorporation into C3 convertase (C3bBb); therefore, it was called C3 nephritic factor (C3NeF) (11, 12). After that, C3NeF was characterized as IgG and IgM autoantibodies with the capacity to recognize neoepitopes of the assembled AP C3 convertase (13–18) (Figure 2A). Studies focused to characterize C3NeF showed that IgG with stabilizing capacity mostly belonged to IgG1 and IgG3 subclasses (14, 15). Moreover, the Fab portion of the immunoglobulin retained the majority of the stabilizing function, although other studies show that the Fc portion also contributes to its functional activity (18, 19).

Several studies have reported that function of C3NeF is dependent on the presence of P. These P-dependent C3NeFs are responsible for activation of the C5 convertase, as reflected by the reduced levels of terminal complement components, and higher sC5b-9 complexes (20–23). In contrast, patients with P-independent C3NeF are characterized by low C3 levels and normal levels of component of the terminal pathway (22, 23). In a recent study, Marinozzi et al. have designed a novel functional assay that allows the identification of patients with autoantibodies with the ability to stabilize the C5 convertase of the AP, which are called C5 nephritic factors (C5NeF) (Figure 2B) (24). A review of previous reports establishes that this autoantibody could be identical to the P-dependent C3NeF described by Tanuma et al. (22), Paixão-Cavalcante et al. (25) and in several previous papers (20, 21). C5NeF has been detected as a unique nephritic factor (10% of cases) or as coexisting with C3NeF (39% of cases) (24). This new terminology is useful for distinguishing NFs in relation to their ability to stabilize C3 or C5 convertases.

All C3NeFs increase the half-life of C3 convertase, because they interfere with the accelerated decay by FH, DAF, and CR1 (25–28). An interesting study used a combination of ELISA and western blot assays to show the heterogeneous control of C3NeF on C3 convertase stabilization against spontaneous and accelerated decay (29). The authors demonstrated that some C3NeFs strongly stabilized the C3 convertase by preventing both spontaneous and FH-mediated decay in the absence of P. Other C3NeFs, however, need P to prevent C3 convertase dissociation (29). Regarding their epitope specificity, most C3NeFs recognize a neoepitope on the assembled C3bBb complex. However, autoantibodies against C3b and FB have recently been reported in patients with C3G, and these have the same capacity to increase the C3 convertase half-life as C3NeF (30–32). The main features for C3NeF and C5NeF are summarized in Table 1.

**Table 1:** Specificity and clinical associations of nephritic factors.

	**Epitope**	**Clinical association (frequency)**
C3NeF	Neoepitope on assembled C3 convertase of the AP (C3bBb)	C3GN (40–50%) and DDD (70–80%) (7, 24, 25, 33, 34) IC-MPGN (40–50%) (34, 35) APL (70-80%) (36, 37) SLE^*^
C4NeF	Neoepitope on assembled C3/C5 convertase of the CP/LP (C4b2a or C4b2aC3b)	C3G and IC-MPGN (3–9%) (38–40) SLE^*^
C5NeF	Neoepitope on assembled C5 convertase of the AP (C3bBbC3bP)	C3GN (67%) and DDD (33%) (24)

AP, alternative pathway; CP, classical pathway; LP, lectin pathway; C3G, C3 glomerulopathy; DDD, dense deposit disease; IC-MPGN, immune complex-associated membranoproliferative glomerulonephritis; APL, acquired partial lipodystrophy; SLE, systemic lupus erythematosus; C3GN, C3 glomerulonephritis

* *The frequency of C3NeF and/or C4NeF in SLE has not been thoroughly investigated*.

### C4 Nephritic Factor (C4NeF)

C4NeFs are autoantibodies that recognize and stabilize the CP/LP C3 convertase (C4b2a) (Figure 2C), increasing its half-life in the fluid and solid phase from a few minutes to several hours (41–44). They were first described in 1980 in one patient with post-infectious glomerulonephritis who had low C3 and C5 levels (41), and in 2 lupus nephritis patients (42). C4NeFs were detected in a patient with chronic glomerulonephritis (44), and in two MPGN patients with very low C3 and C5 levels who also presented C3NeF autoantibodies (45). Coexistence of both C3NeF and C4NeF in the same patient was corroborated upon screening of 100 MPGN patients, 10 of whom presented C3NeF and C4NeF, and reduced C3 and C5 levels (38). The reduced C5 levels observed in these patients is explained by the additional capacity of some C4NeFs to stabilize the C5 convertase and prevent its decay (46).

C4NeFs frequency could be underestimated. As assays for detecting C4NeFs are similar to the assays for detecting C3NeFs (see section Clinical Associations), the generation of C3 activation fragments could be erroneously attributed to the presence of C3NeF instead of C4NeF; to avoid this, data on the levels of C3, C5 and terminal components in the patient's plasma should be known. Thus, C3 levels are lower in C3NeF positive patients than in C4NeF positive patients, while in C4NeF positive patients reduced levels of C5 and most terminal complement components are observed (38).

C4NeFs are most likely of the IgG3 subclass (41). *In vitro* studies with patients' purified IgG have been used to determine the molecular mechanisms of convertase stabilization by C4NeFs. Protection against C4BP-mediated decay was observed (47), and later on confirmed in another study that also showed increased resistance to spontaneous decay and to the proteolytic inactivation of C4b within the C4b2a complex (43). Resistance of C4NeF-C3 convertase to the dissociation induced by CR1 has also been shown (48). C3 and C5 convertases stabilized by C4NeF are strongly resistant to DAF-mediated decay; however, neither C3NeF nor C4NeF allow assembly of the C3 convertase in the presence of DAF (27). A recent study with C4NeFs purified from C3G patients (39) confirms the increased protection against C4BP- and CR1-mediated decay, as well as stabilization of the C5 convertase. Therefore, C4NeF seems to be a highly effective shield against the spontaneous and regulator-induced dissociation of the CP C3/C5 convertases (Figure 2C). The main features for C4NeF are summarized in Table 1.

## Diagnostic Tools to Detect Nephritic Factors

**Figure 3:**
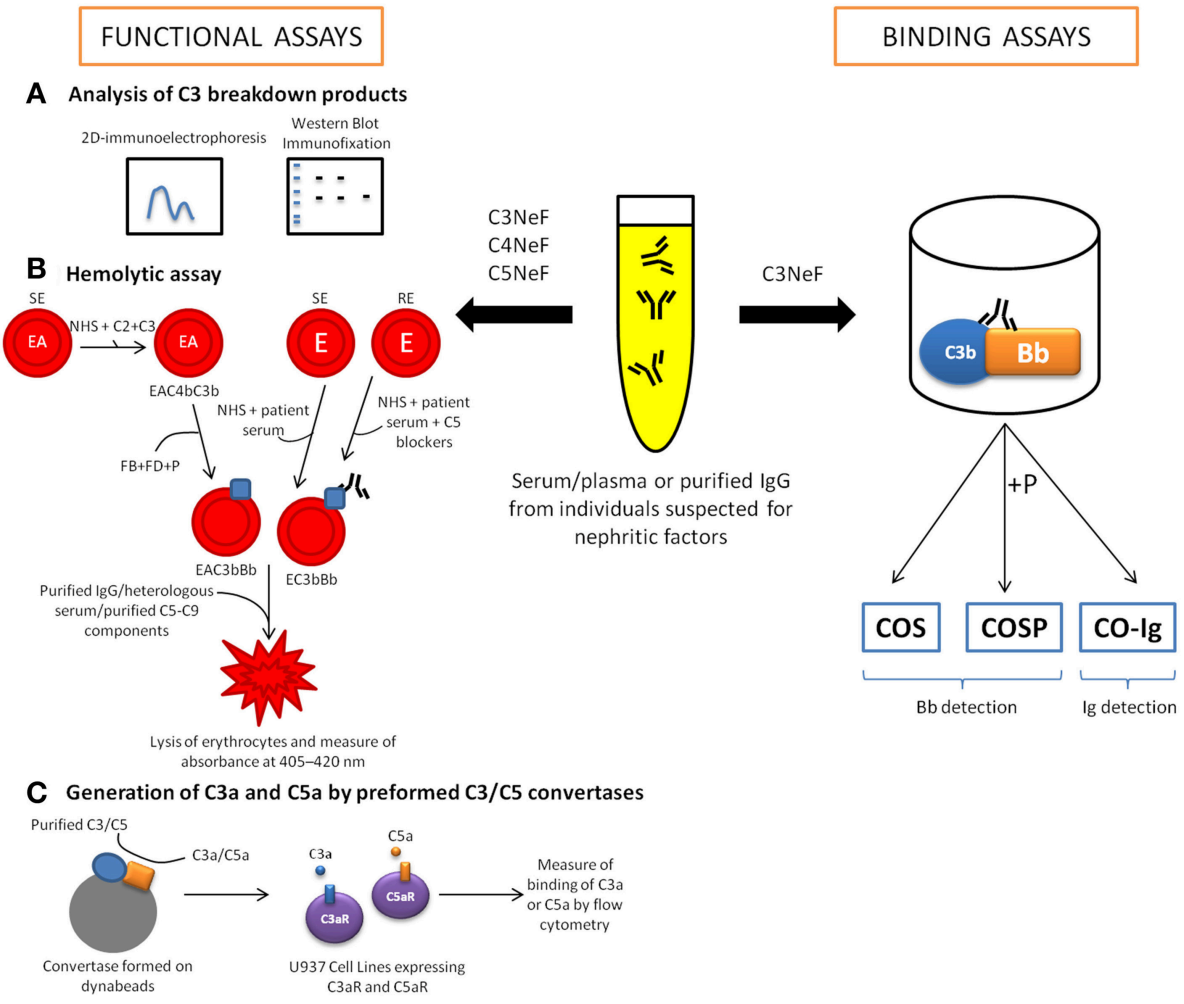
Diagnostic tools for the detection of nephritic factors (NFs). The functional activity of C3NeF can be determined through quantifying complement activation products (mostly C3 fragments) by two-dimensional immunoelectrophoresis, immunofixation electrophoresis or western blotting **(A)**. However, the main tools for the detection of NFs activity are the hemolytic assays, which measure the lysis of sheep (SE)/rabbit erythrocytes (RE) **(B)**. In these assays, purified complement components are added to perform convertases on the surface SE sensitized with anti-sheep antibodies (EA). Later, purified FB, FD and P are added to form specifically alternative pathway convertase. Other assays are developed using whole serum (patient's serum and normal human serum (NHS), in ratio 1: 1) to generate convertases on the SE or RE surface. Considering that RE could be lysed by human serum, terminal pathway needs to be blocked using C5 blockers (OmCI or eculizumab). Another option to measure NF activity is through the quantification of anaphylatoxins, C3a and C5a **(C)**. There are binding assays to detect C3NeF based on enzyme-linked immunosorbent assays (ELISA) that detect immunoglobulins bound to the preformed convertase complex (CO-Ig). Other versions of the ELISA-based binding assay are the so-called C3NeF stabilization assays, which use a polyclonal antibody to detect plate-bound Bb fragment generated either in the absence (COS assay) or in the presence (COS-P assay) of P.

Several methods for the evaluation of NFs have been reported in the literature. Although the old and very simple methods based on mixing normal and hypocomplementemic serum from suspected individuals and the subsequent identification of complement activation markers are still in use, they appear to be of low sensitivity; therefore, a number of more sophisticated protocols have been developed (25). Modern methods are based on measuring the binding of NFs to the pre-formed C3 convertase (see section Binding Assays), or measuring C3/C5 convertase activity in the presence of an NF-suspected sample (see section Functional Assays) (Figure 3). However, such methods represent a substantial challenge due to the labile nature of the C3/C5 convertases, and to a number of situations that mimic NF activity; like as presence of gain-of-function mutations in C3 and FB (49, 50). Apparently, detection of NFs remains problematic, because the 2015 European quality assessment revealed that only half of the participating laboratories properly identified C3NeF reference samples (51). Of note, there is no ideal test capable of covering all difficulties, and both binding assays and functional assays have advantages and drawbacks. Therefore, the combination of convertase assays helps not only improving the specificity of detection but also shedding light on the nature of NFs.

### Binding Assays

Binding assays use convertases reconstituted from purified components on artificial surfaces such as microplates or biosensor chips. Identification of the ligand bound to the convertase complex can give rise to a diagnosis of NF. However, ligand binding may not have any influence on enzyme function, and therefore the positive results of binding assays might not be physiologically relevant. This possibility is reflected by the fact that a certain portion of C3NeF-positive samples detected by binding assays were negative in other functional assays, possibly representing false-positive results (25). However, binding assays performed on purified convertase components allow study the kinetics of convertase dissociation, especially when performed with surface plasmon resonance techniques (52). There are several procedures described for the detection of C3NeF (53). Typically, C3b is immobilized on the microplate surface and then reacted with FB and FD. High yields of microplate-attached alternative convertase complexes are achieved by the addition of NiCl_2_ (53) or NiSO_4_ (25), which outperforms magnesium ions normally supporting convertase formation. The most popular binding assays are enzyme-linked immunosorbent assays (ELISAs) to detect immunoglobulins bound to preformed convertase complex (CO-Ig) (Figure 3). However, such assays might not identify the non-Ig C3 activating factors (54). Another version of the ELISA-based binding assay, the so-called C3NeF stabilization (COS) assay, detects plate-bound Bb fragments with a polyclonal antibody (Figure 3) (25). The COS assay, like assays detecting convertase-bound Igs, begins with C3b deposition followed by the addition of test serum or purified Ig fraction together with FB, FD and optionally, P (COS-P assay) (Figure 3). Detection of Bb fragments on the plate surface coated with C3b indicates the presence of complete AP convertase, which normally decays within minutes when no stabilizing agent is present. Importantly, experimental data by Paixão-Cavalcante et al. showed that 36 out of 101 samples collected from patients with complement-related renal diseases were found to be positive for C3NeF in a COS-P assay but only 16 out of these 36 were positive in a COS assay (25). This difference indicates that C3NeF are very heterogeneous with regard to binding sites and that P might play a role in AP convertase stabilization by C3NeF.

### Functional Assays

#### Analysis of C3 Breakdown Products

As mentioned earlier, the first functional assays relied on mixing control and NF-suspected sera, and further analysis of complement activation products (mostly C3 fragments) by two-dimensional immunoelectrophoresis (55), immunofixation electrophoresis (56) or western blotting (25) (Figure 3). The idea behind these assays is that the spontaneous breakdown of C3 that normally initiates the AP is augmented by convertase stabilization by NFs. Therefore, 3 h of incubation at 37°C is enough time to observe substantial C3 cleavage in samples containing C3NeF in contrast to samples containing normal serum only. Of note, all samples found positive in assays measuring C3 breakdown products were also positive in COS-P binding assays (however, COS-P assays detected more positive samples) but some of them were negative in CO-Ig binding assays (25). A possible explanation is that COS and COS-P assays can be considered quasi-functional assays that detect protein fragments resulting from convertase activity. The same applies to assays performed with surface plasmon resonance techniques, which monitor the association and dissociation of convertase complexes.

#### Hemolytic Assays

Many functional convertase assays measuring the effect of enzymatic activity use red blood cells because they provide an easy readout: the release of hemoglobin, which can be easily quantified by measuring absorbance at 405–420 nm (Figure 3). The general rule is that after deposition of active convertases, the addition of a heterologous serum (e.g., guinea pig serum, which is capable of MAC formation on human convertases but offers limited activity of regulatory proteins) or purified terminal pathway components diluted in ethylene-diamine-tetraacetic acid (EDTA)-gelatin veronal buffer will generate lytic sites. Chelating of magnesium and calcium by EDTA avoids assembly of new convertases but does not influence MAC formation on a platform of already assembled convertases. Depending on the version, these functional assays can analyze purified components (e.g., protein A/protein G-purified Ig fraction from individuals suspected for NFs, which is added during or just after the convertase formation step) or whole serum. The use of whole serum may introduce confounding factors like soluble convertase inhibitors, which can be present at different concentration in particular individuals. Nonetheless, the presence of NF usually prevents activity of such inhibitors and should be manifested in prolonged convertase half-life. Therefore, the assay performed with whole serum as an analyte is a rather quick screening method only showing probability of NF presence, which should be further confirmed by testing of purified Ig fraction.

##### Assays using C3/C5 convertases assembled from purified components

The stepwise building of convertase complex on the surface of sheep erythrocytes enables the analysis of CP/LP and AP enzymes. In the first step, C1 complex is deposited followed by C4, which binds covalently to the membrane. Then, C2 together with the test sample is added, and after incubation the mixture is washed and replaced by guinea pig or rat serum (fully compatible with human serum but of higher terminal pathway efficacy) in EDTA-buffer (39, 46, 57, 58). This experimental design enables the measurement of CP/LP C3 convertase activity, whereas the addition of C3 molecules together with C2 enables the formation of C5 convertase. CP/LP C5 convertase is also formed to deposit AP convertases. Covalent binding of C3b is followed by the removal of noncovalently bound C1 and C2 by prolonged incubation in 10 mM EDTA-buffer. Later, FB, FD, and P are added, thus forming AP convertases (57, 58). After this step, purified Igs purified from patients are used to evaluate NF capacity. This method cannot ensure a clear distinction between C3 and C5 AP convertase activity, but such attempts can be performed either by titrating C3 and thus manipulating C3b density (59) or by developing lytic sites by mixing purified C5-C9 components ± C3 (57). An alternative method of C3b deposition is incubation of erythrocytes with C3 and trypsin at 22°C (59, 60).

##### Functional assays with whole serum

The stepwise formation of convertases from isolated complement components allows the analysis of the purified Ig fraction from an NF-suspected individual, but it also presents certain limitations. A major drawback is the inability to reproduce the physiological serum context, where many proteins are potentially capable of influencing convertases' activity. Therefore, convertase-interacting components identified in assays based on purified complement proteins might not accurately reflect physiological interactions. Convertase assays performed with whole serum are based on a similar design, i.e., convertase formation on erythrocytes as the first step, and generation of lytic sites by addition of EDTA-serum in the second step. Sheep erythrocytes sensitized with anti-sheep antibodies (e.g., amboceptor) offer the possibility of forming CP/LP C3 convertase (C4b2a) when erythrocytes are incubated in C3-depleted serum (61). The complement cascade proceeds up to the step of C3 convertase but no further. Similarly, incubation in C5-depleted serum results in the formation of C5 CP/LP convertase C4b2aC3b (61). The high efficacy of CP/LP convertase formation on sensitized sheep erythrocytes enables the application of low serum concentrations (<1%), excluding the possibility of AP convertase formation, which demands a higher (2%) serum content. Rabbit erythrocytes, which spontaneously activate AP when incubated with human serum, are used for assays of AP convertase. However, this method is unable to distinguish between C3 and C5 convertases, given that both of them contain C3b. Carrying out the reaction in ethylene glycol-bis(β-aminoethyl ether)-*N*,*N*,*N*′,*N*′-tetraacetic acid (EGTA)-buffer excludes the activation of CP/LP, which demands calcium and magnesium, whereas AP only requires magnesium (not affected by EGTA).

The idea of blocking the complement cascade at the level of C5 gives rise to another version of functional convertase assays that can be performed in whole serum, e.g., collected directly from the patient. To this end, C5 inhibitors such as eculizumab or *Ornithodoros moubata* complement inhibitor (OmCI) have been successfully applied (49). Testing whole serum excludes a precise diagnosis of NFs, but it is a useful screening test for searching any components that interfere with convertase activity and/or stability. Recently, this assay was successfully validated in a cohort of C3G patients. The results revealed that the combined analysis of patient's serum and purified Ig fraction properly distinguished patients with convertase-stabilizing activity localized in the Ig fraction (with positive readouts in serum and Ig fraction) from those with familial aHUS caused by gain-of-function mutations in complement components (positive readout only in serum) (62). This method was also successfully applied to diagnose C4NeF in a C3G patient who did not present any other abnormalities in complement proteins and was negative for C3NeF and other autoantibodies against AP components (40). Technically, convertase assays with whole patient's serum demand mixing of patient's sample with normal human serum, usually in 1:1 ratio. Since NF presence is often associated with hypocomplementemia, low content of complement components in unmixed patient's serum results in low amplitude and delayed peak of convertase activity, which may mask convertase-stabilizing effect. However, addition of normal human serum as a source of complement components to NF-containing, hypocomplementemic serum, allows readouts for convertases of prolonged / enhanced activity (49). It is worth mentioning that the first hemolysis-based, two-step assay intended to diagnose C3NeF in patient serum was described more than 30 years ago (63) and was later modified (64, 65). The so-called Rother assay is still in use in some diagnostic laboratories but it often gives inconclusive results. This test uses sheep erythrocytes, which are not an optimal target for AP activation. The kinetics of this process are slower than for analogous AP activation on rabbit erythrocytes, and therefore it is theoretically possible to extract a time point when convertases are already formed but MAC is not yet assembled. In practice, choosing such a time point is arbitrary and will not necessarily work for every individual serum tested. Indeed, many samples cause hemolysis in the first step of this assay, and thus preclude a reliable diagnosis.

An emerging problem for functional convertase assays in detecting NFs is the lack of widely available standards. The most commonly used strategy is to compare results obtained with the results of a reference serum (a highly-positive sample), which has limited availability. This problem is combined with the batch-to-batch differences in erythrocytes, giving rise to high inter-assay variability, and the inability to strictly compare the values obtained in two different laboratories. A possible solution is to use gain-of-function mutants of convertase-forming proteins, which mimic the effect of NFs. For example, a recombinant FB K323E mutant has been proposed as a reference for C3NeF detection (50).

## Generation of C3a and C5a by Preformed C3/C5 Convertases

Another option for measuring convertase activity is to quantify the anaphylatoxins C3a and C5a, which are products of C3 and C5 cleavage by the C3 and C5 convertases, respectively. In this assays, the formation of convertases from purified complement components takes place on the surface of beads. Thereafter, the substrates (C3 or C5) are added together with the tested sample, and the supernatant containing anaphylatoxins is transferred to a culture of reporter cells, previously transfected with C3aR or C5aR. The intensity of the readout in calcium mobilization assays is proportional to the amount of C3a and C5a generated; therefore, this assay enables to distinguish between C3 and C5 convertase activities (66). Such method, not yet demonstrated usefulness for NF detection would be compatible with purified Ig fraction as an analyte.

## Clinical Associations

### C3 Glomerulopathy/Membranoproliferative Glomerulonephritis

C3G is a recently adopted term to refer to a few rare kidney diseases caused by dysregulation of the AP, and characterized by significant C3 deposition in the glomeruli with minimal or no presence of immunoglobulins (67–69). Although there is no a specific histological pattern, most of the C3G cases correspond to some types of the previously denominated MPGN. C3G is classified into Dense Deposit Disease (DDD) and C3 glomerulonephritis (C3GN) on the basis of the electron microscopy findings. Dense Deposit Disease is characterized by distinctive highly electron-dense intermembranous deposits whereas C3GN is characterized by mesangial, intramembranous, subendotelial and sometimes subepithelial deposits (67–72).

C3NeF has been detected mainly in patients with immune complex-associated-MPGN (IC-MPGN) (40–50%) and in patients with C3G (40–80%) (7). Approximately 80% of patients with DDD and in 40%-50% of patients with C3GN (51, 72) are positive for C3NeF. Recently, Donadelli et al. published a study providing an interesting method to detect and characterize C3NeF in patients with C3G and IC-MPGN (29). The authors reported that most of the patients with DDD had C3NeF that stabilized C3 convertase in the absence of P, while in the remaining patients (C3GN and IC-MPGN) C3NeF was P-dependent (C5NeF), resulting in both C3 and C5 convertase dysregulation (29). These results are reliable with the previous observation that C3NeF targets both C3 and C5 convertases in 67% of patients with C3GN (24), and that terminal pathway dysregulation is significantly higher in C3GN than in DDD (24, 29). Moreover, our group documented that a subset of C3G patients with low P levels (most of them C3GN) was characterized by C5 consumption and high sC5b-9 levels in plasma (33). However, although our results are consistent with the presence of C5NeF, we could not prove it due to the lack of a specific assay to detect this autoantibody.

The pathogenic role of C3NeFs in C3G has been debated for a long time. Although C3NeFs are closely linked with C3G, they have been detected in healthy individuals, suggesting that this autoantibody is part of the normal immune repertoire (73, 74). Therefore, a fundamental unanswered question is whether C3NeF is the cause of the disease, or whether C3NeF is a consequence of the disease process that then acts exacerbating the disease pathology (17, 74, 75). Moreover, plasma C3 consumption and disease severity do not always correlate with the presence and activity of C3NeF (76–78). Autoantibodies related to complement components, such as anti-FB, anti-C3b, anti-FH or other autoantibodies (e.g., ANAs, ANCAs or cryoglobulins) have been detected in smaller percentages of patients (79). Overall, in the series from Mayo Clinic, 13.4% of C3G patients were positive for autoantibodies other than C3NeF (80).

C4NeFs autoantibodies have been mainly found in patients with MPGN (38, 44, 45, 81). A study in 100 patients with hypocomplementemic MPGN showed that as many as 19 patients had C4NeF (alone or in combination with C3NeF) (38). The 10 patients having both C3NeF and C4NeF presented low C3, C5, and terminal complement component (C6–C9) levels, as well as massive C3 deposits in the kidneys by immunofluorescence microscopy; moreover, they presented therapy-resistant hypocomplementemia, a higher incidence of nephritic syndrome and a poorer prognosis than patients positive for only C3NeF or C4NeF. These 10 patients would have been diagnosed of C3G according to current criteria (70). Another interesting observation in this study is that one patient was subsequently C3NeF positive, C3NeF negative, and C4NeF positive, suggesting than autoantibody specificity can change along disease evolution.

More recently, a C4NeF that reduced decay of the CP C3 and C5 convertases was detected in 1 out of 13 C3G patients (40). The patient had reduced C3 levels, but C4 and FB levels were normal. C4NeFs have been also found in 4/100 C3GN patients, and in 1/68 DDD patients, representing 3% of the 168 C3G patients included in the study (39); 1 of these patients also had anti-FH autoantibodies, and another patient had C3NeF. The low frequency of C4NeF-positive patients (3%) in comparison with C3NeF-positive patients (52%) in this large and well documented C3G series suggests that the actual contribution of C4NeF to the pathogenic mechanism is less relevant than the contribution of C3NeF, and it could be associated with an infectious trigger. This possibility is in line with the authors' observation that kidney biopsies from their C4NeF-positive patients are suggestive of post-infectious glomerulonephritis which evolved into C3G.

### Acquired Partial Lipodystrophy (APL)

APL or Barraquer-Simons syndrome (ORPHA:79087) is an ultra-rare disease characterized by progressive loss of adipose tissue starting from the face and then extend to the neck, shoulders, upper extremities and then to the thorax. Patients may accumulate fat excess in the lower extremities after puberty, especially female patients (36). APL is more frequent in females than males (4:1) (82). Metabolic syndrome and its comorbidities are absent or infrequent in this syndrome (83). However, autoimmune diseases, particularly SLE and dermatomyositis, have been reported in patients with APL (36, 84).

Adipsin is an adipokine produced by mature adipocytes (85). In 1992, White et al. shown that human adipsin was identical to complement FD and that it major source was located in the adipose tissue (86). Moreover, adipocytes also express C3, FB and complement regulators such as P and FH, and consequently complement is connected to adipocyte biology (87). Indeed, complement system functions on adipose tissue, as preadipocyte differentiation and triacylglyceride synthesis, have been described (88).

C3 hypocomplementemia in relation with the presence of C3NeF have been reported in almost all patients with APL (82–84, 89–103). Therefore, as a consequence of the presence of C3NeF, approximately 20% of patients with APL develop C3G in a period of 8 years after the onset of lipodystrophy, and some of them evolve to end-stage renal disease requiring renal transplantation (83). Although complement dysregulation is a common mechanism in C3G and APL, the exact mechanism of fat loss remains unclear (87). There is one experimental study which described complement-mediated lysis of adipocytes *in vitro* by C3NeF (87, 104). However, the proposal fails to answer the key question of not all patients with C3NeF develop lipodystrophy.

### SLE and Infections

C3NeF have been described in patients with SLE, associated in most cases with partial lipodystrophy and/or DDD (90, 95, 96, 105–111). However, there are several studies describing patients with lupus nephritis, complement abnormalities and the presence of autoantibodies against AP proteins in absence of C3NeF (112–114). For example, our group published a case of lupus nephritis with autoantibodies against C3, FB and P, which was negative for C3NeF (112). Other authors reported that approximately 30% of patients with lupus nephritis were positive for anti-C3 autoantibodies (113). These autoantibodies have a similar effect as C3NeF, which should be taken into consideration during the diagnosis.

C3NeFs have been detected in patients with meningococcal infections, without renal impairment (115–121). By contrast, there are several cases series of post-streptococcal acute glomerulonephritis associated with C3NeF activity (122–125).

C4NeFs have also been found in a few SLE patients (42), and occasionally following streptococcal (41) or meningococcal (46) infections. The latter is a peculiar case of a C4NeF autoantibody that did not provoke a renal phenotype, but it was found in a patient with severe meningococcal disease. This C4NeF autoantibody stabilized the C3 and C5 convertases from the CP and generated C3 deficiency by increased consumption (46).

## Diagnostic Approach to Identify Nephritic Factors

Several renal diseases are caused by complement dysregulation, and while the most common causes of impaired complement regulation are pathogenic mutations or anti-FH autoantibodies in atypical hemolytic uremic syndrome (aHUS), in C3G NFs are the most frequent finding (34, 69). Although definitive diagnosis should be based on light and electron microscopy findings of kidney biopsy, some laboratory tests may support the diagnosis.

After suspicion or definitive diagnosis of C3G, C3NeFs have to be screened in the first steps, together with serum C3 levels. Other parameters included in the current laboratory diagnostic protocol are commented below.

Serum C3 and C4 levels can be measured fast and easily by automated methods, as nephelometry or turbidimetry, but normal C3 levels do not exclude NF presence. Complement activation can also be assessed by measuring C3d, FB, P and sC5b9, not only to demonstrate AP dysregulation but to differentiate between C3/C4NeFs and C5NeF (33, 51, 126).

Serum from C3G patients should be screened for autoantibodies (16), including anti-FH (55, 127, 128), anti-FB (31, 129), and anti-C3 (32), all of them causing alternative pathway dysregulation, but the presence of any of these autoantibodies do not exclude NF existence, as anti-FH autoantibodies are frequently associated with C3NeF in pediatric C3G patients (130). FH deficiency is associated with DDD and C3GN (131, 132), as it leads to totally uncontrolled AP activation. Besides serum FH levels, a genetic study is necessary because single point mutations in FH gene causing C3GN have been described (102, 133).

Genetic screening of AP regulators and convertase components should be undertaken in a complete complement study of C3G patients. Common and pathogenic variants associated with C3G have been described in FH, FI, FB, CD46, C3 and thrombomodulin genes, influencing on long-term outcome (5, 34, 35). Again, the presence of any mutation or variant do not exclude NF existence, as it's shown in a French C3G series, where C3NeF was present in 50% of patients carrying mutations in complement genes (34).

Defective complement regulation has been demonstrated in *C3* and *CFB* mutations associated with C3G; these mutations impair C3b inactivation by FI (134) increase convertase resistance to dissociation by FH (135), or promote high–affinity binding of C3 to FB (136).

A genetic and protein approach to study the Factor H-Related (FHR) proteins is also recommended, as mutations and gene rearrangements causing FH deregulation have been reported over the last years in C3G patients (137–143).

## Management and Therapeutic Perspectives for Patients With Nephritic Factors

In the last years, there has been an important progress in the knowledge about treatments for autoimmune diseases. The presence of NFs should be considered the main autoimmune cause of diseases as C3G and IC-MPGN. In most of consensus reports, the experts indicate that treatment is directed to improve clinical parameters. However, a specific disease-directed treatment for patients with NFs has not been established. There are no randomized trials able to generate therapeutic decisions, so all current recommendations are based on low-quality evidence since the patients' series include a heterogeneous and limited number of cases. Therapies are divided into common treatments indicated to all autoimmune diseases and specific complement blocking drugs:

- Plasma Exchange Therapy/Plasmapheresis: In order to remove NFs, plasma exchange has been used with irregular efficacy. A combined treatment based on plasma exchange and immunosuppressants has proved useful in patients with C3G positive for C3NeF (144). However, with these results it was difficult to evaluate the effect of plasma exchange since the treatments were administered all together. An interesting work showed that using long-term plasmapheresis removed C3NeF activity from the serum of patients with DDD (145).
- Immunosuppressive Therapy: These treatments are focused to limit and/or reduce the production of NFs, and they have mainly been proved in patients with C3G and MPGN. The benefit of long-term alternate-day steroid therapy for idiopathic MPGN has been suggested; however, these studies include a combination of patients with different types of MPGN, limiting possible conclusions (75, 146, 147). An interesting report from the Spanish Group for Glomerular Disease Study showed the effectiveness of the combination of mycophenolate mofetil (MMF) and glucocorticoids in patients with C3GN (148). This study compared the clinical evolution of three groups of C3GN patients: one without glucocorticoids treatment, one with glucocorticoids but no MMF and another one based on MMF plus glucocorticoids. The results showed that eight out of ten patients with C3NeF (80%) were more likely to achieve remission with this treatment. However, this clinical improvement could not be attributed to C3NeF disappearance because it determination was not performed in all patients during the whole course of the treatment. A similar although less impressive response was described in a large cohort from the Mayo Clinic (80).
- Monoclonal Antibody Therapy: Antibody-producing B cell-targeted therapies should be effective in patients with C3NeF, but the data using a monoclonal anti-CD20 antibody (rituximab) have not been consistent regarding C3NeF activity and clinical response (149–151). The experience on the use of rituximab in MPGN, C3GN, and DDD with or without NFs is limited to case reports and retrospective case series. Patients with IC-MPGN who were treated with rituximab showed partial and complete responses in the majorities of cases, while treatment was not effective in most C3GN and DDD cases (152).
- Complement Directed Therapies: Complement blocking agents such as eculizumab (Soliris) have been also tried in patients with NFs as the only pathogenic cause or in combination with mutations in complement components (153, 154). Almost all authors coincide in that eculizumab therapy should be considered after the failure of immunosuppressive and plasma exchange treatments to improve renal function. A large series using eculizumab in C3G have been published by Le Quintrec et al. from France (154). Briefly, 23% of their patients had a global clinical response, while 23% had a partial response and the remaining 54% were nonresponders. The patients with a good clinical response had lower glomerular filtration, a more rapidly progressive course and more extracapillary proliferation as determined by kidney biopsy (154). Parameters including age, extent of renal fibrosis, nephrotic syndrome, C3 levels, C3NeF activity, sC5b-9 concentration or pathogenic complement gene variants did not differ between responders and nonresponders (154). There are several reports that show that eculizumab may be a specific and useful treatment in C3NeF-related DDD (103, 151). A potential benefit of eculizumab treatment was suggested for the five C4NeF-positive patients described by Zhang et al. (39), who presented elevated plasma levels of sC5b-9.

New complement modulators targeting C3 convertase activity, such as soluble CR1 (sCR1), have been proved in sporadic cases of C3G (155). The *in vitro* activity of sCR1 prevents the hemolysis of rabbit erythrocytes caused by C3NeF with greater effectiveness than FH. The administration of sCR1 to Cfh–/–/huCR1-Tg mouse resulted in markedly reduction of C3 deposits along the glomerulus. However, C3 staining not changed before and after treatment in an 8-year-old girl positive for C3NeF with biopsy-proven DDD although the authors observed a transient improvement in C3 serum levels and a decreased of sC5b-9.

## Author Contributions

All authors listed have made a substantial, direct and intellectual contribution to the work, and approved it for publication.

### Conflict of Interest Statement

The authors declare that the research was conducted in the absence of any commercial or financial relationships that could be construed as a potential conflict of interest.

## Abbreviations

AP: alternative pathway
CP: classical pathway
LP: lectin pathway
FB: Factor B
P: Properdin
FH: factor H
FI: Factor I
NF: nephritic factor
C3NeF: C3 nephritic factor
C4NeF: C4 nephritic factor
C5NeF: C5 nephritic factor
C3G: C3 glomerulopathy
DDD: dense deposit disease
IC-MPGN: immune complex-associated membranoproliferative glomerulonephritis
APL: acquired partial lipodystrophy
SLE: systemic lupus erythematosus
C3GN: C3 glomerulonephritis.
